# Efficacy and Satisfaction of a Chemically Characterized *Malva sylvestris* L. Extract-Based Food Supplement for Functional Constipation in Healthy Consumers: Preliminary Results of a Questionnaire-Based Survey

**DOI:** 10.3390/nu17010077

**Published:** 2024-12-28

**Authors:** Lorenza Francesca De Lellis, Hammad Ullah, Maria Vittoria Morone, Daniele Giuseppe Buccato, Alessandra Baldi, Alessandro Di Minno, Danaé S. Larsen, Roberto Sacchi, Maria Daglia

**Affiliations:** 1 Department of Pharmacy, University of Napoli Federico II, Via D. Montesano 49, 80131 Naples, Italy; lo.delellis2@gmail.com (L.F.D.L.); hammadrph@gmail.com (H.U.); d.buccato@gmail.com (D.G.B.); alessandra.baldi.alimenti@gmail.com (A.B.); alessandro.diminno@unina.it (A.D.M.); 2 Department of Experimental Medicine, Section of Microbiology and Clinical Microbiology, University of Campania “L. Vanvitelli”, 80138 Naples, Italy; mariavittoria.morone@unicampania.it; 3 CEINGE-Biotecnologie Avanzate, Via Gaetano Salvatore 486, 80145 Naples, Italy; 4 School of Chemical Sciences, The University of Auckland, Auckland 1010, New Zealand; 5 Applied Statistic Unit, Department of Earth and Environmental Sciences, University of Pavia, Viale Taramelli 24, 27100 Pavia, Italy; roberto.sacchi@unipv.it; 6 International Research Center for Food Nutrition and Safety, Jiangsu University, Zhenjiang 212013, China

**Keywords:** *Malva sylvestris* L., food supplement, functional constipation, consumer questionnaire-based survey

## Abstract

Background/Objectives: *Malva sylvestris* L. is rich in mucilage and is traditionally used for the management of numerous ailments including gastrointestinal disorders. Functional constipation (FC) is a gastrointestinal condition characterized by defecation anomalies such as infrequent stools, difficulty in stool passage, or both in the absence of pathological abnormalities. FC can be reduced through lifestyle factors and dietary intervention. This consumer-based survey aimed to assess the efficacy of a *M. sylvestris* extract-based food supplement on the improvement of FC. Methods: Healthy participants (*n* = 56), enrolled in a consumer-based survey, took a food supplement containing a chemically characterized *M. sylvestris* extract at a dose of 20 mL/day (containing 750 mg of *M. sylvestris* extract rich in food fiber and polyphenols) for 20 days on the advice of their pharmacist. The study evaluated bowel movement frequency (intestinal diary), stool consistency (Bristol Stool Form Scale, BSFS), and abdominal pain (Visual Analogue Scale, VAS), at baseline (T0), after 10 days (T1), and after 20 days (T2). Results: A significant increase in bowel movement frequency and stool consistency (*p* < 0.001) with a significant decrease in abdominal pain (*p* < 0.001) was observed. Additionally, this food supplement was well-tolerated as no adverse effects were reported by the enrolled subjects. Conclusion: *M. sylvestris*-based food supplement showed promising effectiveness and satisfaction in improving FC in healthy subjects, however, randomized clinical studies are needed to confirm these preliminary results.

## 1. Introduction

Constipation refers to a group of gastrointestinal symptoms that include abdominal pain, firm stools, difficult or infrequent bowel movements/deviation from normal frequency, and/or the feeling of incomplete evacuation of stool. Functional constipation (FC) lacks anatomical abnormalities or systemic etiology. It is influenced by environmental factors, stress, and diet [1]. Constipation is primarily self-diagnosed via the self-reporting of symptoms or a symptom-based questionnaire in cross-sectional surveys [2]. Suares and Ford [3] conducted a comprehensive meta-analysis to evaluate the risk factors and prevalence of chronic idiopathic constipation in the community. The global prevalence of chronic idiopathic constipation is 14% under the Rome IV criteria but drops to 6.8% with the Rome III criteria. FC is inversely related to the quality of life of the affected subjects, and is also associated with increased frailty, particularly in elder subjects [4].

Increased dietary fiber and lifestyle changes are first-line treatments, followed by laxatives if necessary [5]. Dietary fiber is not digested in the small intestine; some are fermented by bacteria or can induce bulk formation in the colon, drawing water into the colon [6]. However, the results of systematic reviews indicate that soluble fiber rather than insoluble fiber alleviates symptoms related to constipation [7]. The soluble fiber psyllium has been demonstrated to lessen defecation discomfort and improve stool weight, frequency, and consistency [8]. Research has shown that a low dietary fiber intake (7 g/day) could be associated with a decrease in the frequency of stools, and it is generally advised to gradually increase fiber consumption, up to 30 g per day. In addition, despite the availability of over-the-counter laxatives and dietary modifications, many individuals with FC experience incomplete relief, adverse effects, or poor adherence. For instance, increased fiber consumption could also lead to adverse effects like gas, bloating, and distension [2]. This underscores the need for alternative therapies that are both effective and well-tolerated. Plant-based supplements offer a promising avenue for addressing these challenges by combining natural bioactive compounds with dietary fiber [9].

*Malva sylvestris* L. (Malvaceae) is notable for its many applications and has been consumed since 3000 BC [10]. Also known as common mallow, it is indigenous to Europe, North Africa, and Asia, and has a long history of use in the Mediterranean region as food, and because of its potential therapeutic uses [11]. The leaves, in particular, have antioxidant, anti-inflammatory, and skin tissue integrity properties [11]. Furthermore, recent studies have established anti-ulcerogenic effects, showing the aqueous extract of *Malva sylvestris* L. to be more efficacious than cimetidine, a peptic ulcer and heartburn drug [12]. In addition, it has hepatoprotective properties and a mild laxative effect, suggesting its use for gastrointestinal ailments [11]. *M. sylvestris* is rich in mucilage [13], with the leaves (6–7.2%), flowers (3.8–7.3%), and roots (7.5%) often containing the highest percentages of crude mucilage [14,15,16]. The main components of the mucilage include glucuronic acid, galacturonic acid, rhamnose, galactose, fructose, glucose, sucrose, and trehalose; however, additional components have been identified, including uronic acid, arabinose, mannose, xylose, fucose, raffinose, and 2″-*O*-α-(4-*O*-methyl-α-D-glucuronosyl)-xylotriose [14,15,17,18]. These compounds are believed to improve stool consistency, enhance water retention in the gut, and modulate the gut microbiota, making it a potential therapeutic agent for managing gastrointestinal disorders.

Thus, *M. sylvestris* has been identified as a potential therapy to improve FC via a nutritional intervention of increased dietary fiber intake and accelerated stool intestinal transit. The current study aimed to collect data via a survey of food supplement consumers to assess whether a *M. sylvestris* extract-based food supplement can help with FC. The questionnaire also assessed the frequency of gastrointestinal movements, stool consistency, and a possible improvement to the quality of life through a reduction in abdominal pain, as well as consumers’ acceptance of the food supplement. Moreover, the chemical composition of *M. sylvestris* extract was determined to understand the contribution of dietary fiber and other bioactive compounds present in *M. sylvestris* extract.

## 2. Materials and Methods

### 2.1. Food Supplement

The commercial food supplement Laxamov^®^ (notification number: 171854) (Labomar S.p.A, Istriana, TV, Italy) was utilized in this study. The Italian Ministry of Health was notified of its distribution based on *M. sylvestris* extract (750 mg per 20 mL). The supplement also contains sorbitol (Labomar S.p.A, Istriana, TV, Italy) (10 g per 20 mL) as a food additive (E 420) at a dose lower than that considered responsible for laxative effects (50 g/day) [19,20]. According to the manufacturer’s specifications, this food supplement complies with European specifications for contaminants and microbiological limits.

### 2.2. Total Dietary Fiber Determination

The total dietary fiber (TDF) content of *M. sylvestris* extract (mixed with maltodextrin (1:4) as a carrier) was determined in triplicate in accordance with the manufacturer’s protocol using the TDF Assay Kit (Neogen, Lansing, MI, USA) [21], which represents a simplified version of the official AOAC 985.29 method. The total soluble and insoluble fiber content of *M. sylvestris* extract was determined using a gravimetric method. This involved eliminating starch and proteins through treatments with heat stable enzymes i.e., α-amylase (~55 U/mg), amyloglucosidase (~3260 U/mL), and protease (~6 U/mg of protein). Following these treatments, a precipitate was observed, indicating the presence of insoluble fiber. High-molecular-weight soluble fiber was precipitated using 78% ethanol (Merck Life Science S.r.l., Milano, Italy) and measured using the gravimetric method.

The carrier maltodextrin was also analyzed for dietary fiber content to ensure correction in the calculation of *M. sylvestris* extract fiber content. No dietary fiber was detected in maltodextrin, confirming that the soluble and insoluble fiber was determined to have originated from *M. sylvestris*. Five aliquots of *M. sylvestris* extract (1.000 ± 0.005 g) were incubated at 100 °C in 50 µL heat-stable α-amylase solution to allow starch gelatinization, hydrolysis, and depolymerization. The samples were then incubated at 60 °C with 100 µL protease solution (to solubilize and depolymerize the proteins) and 200 µL amyloglucosidase solution (to hydrolyze the starch fragments into glucose). After incubation, the samples were filtered to separate the insoluble fiber. The supernatant was treated with approximately four volumes of ethanol to precipitate soluble fibers and remove the depolymerized proteins and glucose (from starch). The samples were freeze-dried, and the residue analyzed for proteins using the Kjeldahl method [22], while ash content was determined by incineration at 525 °C. The TDF was calculated using the following formula:

TDF=(Insolublefiber+Solublefiber)−(Protein+Ash)

### 2.3. UHPLC-HRMS Analysis of M. sylvestris Extract

#### 2.3.1. Sample Preparation

The dry extract of *M. sylvestris* with maltodextrin used as a carrier was loaded on to a C18 solid-phase extraction (SPE) column previously conditioned with 5 mL methanol (MeOH; Merck Life Science S.r.l., MI, Italy) and 5 mL H_2_O. After loading, the column was washed with 10 mL H_2_O. The sample was eluted with a 2.5 mL mixture of MeOH:H_2_O (95:5, *v*/*v*) and 1 mL of this solution was transferred to an autosampler vial for ultra-high-performance liquid chromatography–high-resolution mass spectrometry (UHPLC-HRMS) analysis.

#### 2.3.2. UHPLC-HRMS Analysis

UHPLC-HRMS/MS analysis was performed on a Thermo Scientific™ Vanquish™ UHPLC system, equipped with a VF-P10-A binary solvent delivery system, a VC-D11-A photodiode array detector, a VH-C10-A column compartment, and VF-A10-A autosampler. The UHPLC system was coupled online to a Orbitrap Exploris 120 mass spectrometer (Thermo Fisher Scientific, Bremen, Germany) equipped with a heated electrospray ionization probe (HESI II) operating in negative and positive mode. The sample was weighed and solubilized in methanol at a concentration of 100 mg/mL, the sample was vortexed at RT for 30 min and centrifuged at 4 °C for 10 min, and the supernatant was collected and directly injected in UHPLC-HRMS.

Chromatographic separation was performed on a Kinetex^®^ 2.6 µm EVO C18 100 Å, LC Column 150 × 2.1 mm (Phenomenex, Bologna, Italy). The column temperature and the flow rate were set at 40 °C and 0.4 mL/min, respectively. The mobile phases were as follows: H_2_O (A) and ACN (B) both acidified with 0.1% HCOOH (*v*/*v*) with the following gradient: 0.01–2.00 min, isocratic to 2% B; 2.01–22.00 min, 2–95% B; 22.01–24.00 min, isocratic to 95% B; 24.01–25.00 min, 95–2% B; then, column re-equilibration was performed for five minutes.

MS was calibrated by Thermo Pierce™ FlexMix™ (Thermo Fischer Scientific, Waltham, MA, USA) calibration solutions in both polarities. Full MS (100–1500 *m*/*z*) and data-dependent MS/MS were performed at a resolution of 60,000 and 15,000 FWHM, respectively. A Normalized Collision Energy (NCE) value of 30 was used. Source parameters were as follows: sheath gas pressure, 40 arbitrary units; auxiliary gas flow, 15 arbitrary units; spray voltage, +3.0 kV, −2.0 kV; capillary temperature, 320 °C; and auxiliary gas heater temperature, 300 °C. The identification of investigated analytes was carried out by comparing their retention times and MS/MS data with those present in the literature. Data analysis and processing were performed using FreeStyle™ 1.8 SP2 and the commercial software Compound Discoverer v. 3.3.1.111 SP1 (Thermo Fisher Scientific, Bremen, Germany).

### 2.4. Evaluation of Efficacy and Tolerability

#### 2.4.1. Survey Design

A questionnaire-based survey aimed at food supplement consumers was undertaken to evaluate the efficacy and satisfaction of a food supplement based on *M. sylvestris* extract in healthy subjects. The survey was in the form of a questionnaire encompassing an intestinal diary and validated questionnaires including the Bristol Stool Form Scale (BSFS) and a Visual Analogue Scale (VAS). The assessment of the tolerability of the food supplement was performed through evaluation of adverse reactions (ARs) using a form based on that used by the Italian Phytovigilance System (IPS) to report possible ARs after the ingestion of food supplements.

Enrollment was based on subjects who communicated symptoms related to FC to a pharmacist. These subjects were directed to a separate room, where they received an exhaustive explanation of the survey by a pharmacist. The participants underwent three interviews: at recruitment (i.e., at the baseline, t0), after 10 days (t1), and after 20 days (t2). At recruitment, the inclusion of the subjects in the survey was also evaluated by a physician, who validated their eligibility and health prior to participation. After they gave written consent, in addition to the self-completion of questionnaires administered by the physician, each subject was given the form to report possible ARs after the ingestion of food supplements. These forms were collected at the end of the survey (t2). All subjects were suggested to consume the *M. sylvestris* extract-based food supplement (20 mL/day), for 20 days. Any data gathered within this survey were treated in strict accordance with the European Data Protection Regulation (GDPR) and used for research purposes only.

Overall, the survey involved a cohort of 56 subjects with self-reporting FC, who had asked their pharmacist for a food supplement to treat FC and where the commercial *M. sylvestris*-based food supplement was suggested for them. The questionnaire-based survey of subjects was conducted between October 2023 to March 2024. Rome IV Criteria were used to classify subjects with FC and to enroll the subjects in this survey. Individuals who have had symptoms of chronic constipation for at least 3 months (with onset at least 6 months before) and no organic gastrointestinal pathology were classified according to the Rome IV Criteria as having irritable bowel syndrome with constipation (IBS-C), FC, opioid-induced constipation, and functional defecation disorders. Subjects suffering from IBS-C were excluded from the survey, as the focus was on individuals with simple FC, a condition considered non-pathological and for which the use of food supplements represents a more appropriate remedy. The inclusion criteria were subjects of either sex, aged 18 to 75 years, subjects able to understand and sign the informed consent, and subjects with FC and a number of bowel movements less than 2 per week. Exclusion criteria were subjects with IBS-C (according to the Rome IV Criteria), pregnant or breastfeeding females, subjects who regularly take laxatives, subjects suffering from organic intestinal pathologies, subjects who had undergone surgery on the gastrointestinal tract, subjects with gastroesophageal reflux, subjects with Parkinson’s and Alzheimer’s disorders, and individuals who use opioid medications or other medications that have a significant impact on intestinal function (i.e., antidepressants or antacids containing aluminum). Data collected by the physician included the demographics and baseline clinical data of the enrolled subjects.

#### 2.4.2. Primary and Secondary Objectives

The primary objective of this investigation was to evaluate the impact of supplementation with *M. sylvestris* in subjects with FC for the frequency of gastrointestinal movements (bowel movements/week). The number of bowel movements per week was determined via a self-assessment questionnaire, which was inserted into the “intestinal diary” that the subject filled out daily. For the objective to be satisfied, the increase in the number of bowel movements must be at least one per week.

Secondary objectives of this investigation included consistency of stool (which, together with the frequency of intestinal evacuation, has been indicated in the European Medicines Agency (EMA) and European Food Safety Authority (EFSA) guidelines as an indicator of FC) and frequency of characteristic symptoms of the constipated subject, i.e., abdominal pain. The BSFS was used to assess the stool consistency on a spectrum of seven types. Stool types 1 and 2 denote hard or lumpy stools, while stool types 6 and 7 are indicative of loose or watery stools, where subjects with stool consistency type 1 and 2 were characterized as suffering from constipation. The abdominal pain was assessed via a VAS.

Current food regulations permit the use of all the components in the food supplement under study. However, the food supplement consumers were closely observed by the physician and pharmacist and any suspected adverse reactions were reported via the VigiErbe online phytovigilance system, according to the provisions of the Italian Istituto Superiore di Sanità [23].

### 2.5. Statistical Analysis

The sample size calculation was made using a 1-β power value of 0.95 and a significance level α = 0.05. The sample size was determined to be 56 participants.

The objective of the statistical analysis was to compare the score values for the weekly evacuations, BSFS and VAS scales, and the characteristics of the subjects recruited (sex and age). The most suitable statistical analysis for this type of study is a linear mixed model (LMM) with random intercept, in which the score values of the scales is the dependent variable, while the measurements (T0, T1, and T2), sex (two-levels factor), and age (standardized to mean = 0 and SD = 1) of the recruited subjects constitute the independent variables. The interaction sex × measurement was inserted to account for differential responses to measurements between males and females. The identity of the subjects was entered into the model as a random effect to control any differences due to the specific conditions of the persons recruited in the survey. All analyses were carried out in R 4.2.1 [24] using the package lme4 [25].

## 3. Results

### 3.1. Chemical Composition of M. sylvestris Extract

#### 3.1.1. TDF Content

The analysis confirmed that *M. sylvestris* extract contained 2.7 ± 1.1 g/100 g of insoluble fiber and 5.9 ± 0.2 g/100 g of high-molecular-weight soluble fiber, with no detectable dietary fiber in the maltodextrin carrier. The content of dietary fiber in maltodextrin was determined to correct the content of dietary fiber of *M. sylvestris* extract. Dietary fiber was not found in maltodextrin, suggesting that the determined soluble and insoluble fiber was from *M. sylvestris*.

#### 3.1.2. RP-UHPLC-MS Analysis

**Figure 1:**
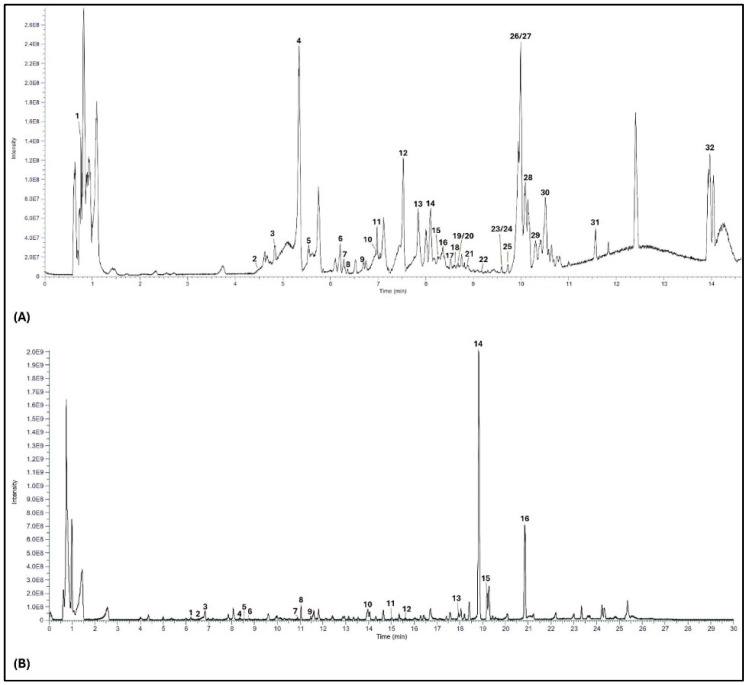
UHPLC chromatograms of *M. sylvestris* extract with UV detection, compounds identified both in negative ionization (**A**) and positive ionization (**B**) mode.

*M. sylvestris* extract was chemically characterized by using a chromatographic method coupled with mass spectrometry [26]. Using negative ionization mode, 32 compounds were identified (Figure 1A and Table 1), including phenolic acids (malic acid, gallic acid, 4-methoxybenzoic acid, caffeic acid, rosmarinic acid, *p*-coumaric acid, 4-hydroxybenzoic acid, and protocatechuic acid), flavonoids (vicenin 2, myricetin-3-hexoside-glucuronide, quercitrin, apigenin-4′-*O*-β-glucopyranoside, gossypetin, myricetin-3-*O*-glucuronide, quercetin-3-*O*-glucuronide, rubone, kaempferol-3-*O*-glucuronide, chysin, fisetin, quercetin, luteolin, kaempferide-3-glucuronide, naringenin, hesperetin, apigenin/genistein, kaempferol, and rhamnetin), and lipids (azelaic acid, 9,12,13-trihydroxy-octadecadienoic acid, 9,12,13-trihydroxy-octadecenoic acid, 1-*O*-palmitoylglyceride, and 9-hydroxy-10(E),12(Z),15(Z)-octadecatrienoic acid). Similarly, under positive ionization mode, we identified 16 compounds (Figure 1B and Table 2), including flavonoids (vicenin 2, quercetin-3-*O*-β-glucuronide, and kaempferol-3-*O*-glucuronide), acetamide (*N*-(9-oxodecyl) acetamide), carotenoids (loliolide), lipids (dehydrophytosphingosine, phytosphingosine, stearidonic acid, lysophosphatidylcholine (LPC; 16:0), 1-*O*-linolenoyl glyceride, oleamide, and erucamide), and chlorophyll derivatives (pheophorbide A).

### 3.2. Survey Among Consumers on the Evaluation of Efficacy and Satisfaction of the Food Supplement

**Figure 2:**
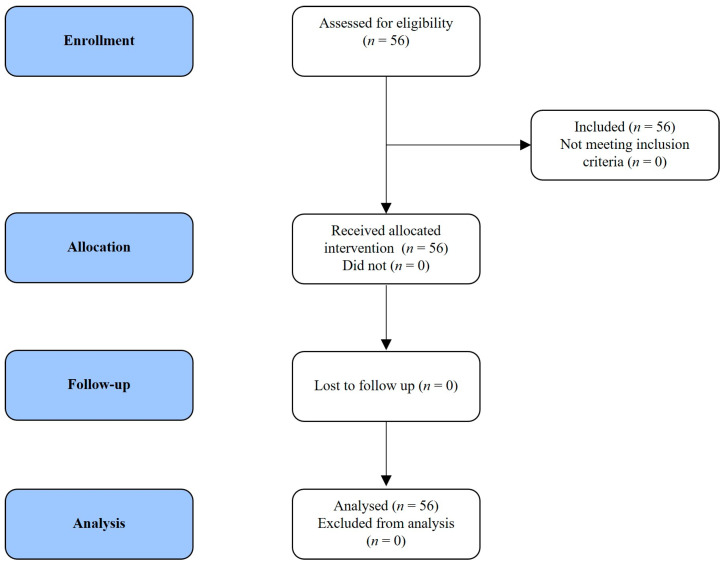
Survey flow chart diagram.

The survey flow chart [27] is shown in Figure 2. The baseline characteristics of the subjects of each group are summarized in Table 3. In total, 56 subjects (12 males and 44 females) were enrolled in the survey, with the mean age for male subjects being 58.1 ± 18.3 years, and 52.2 ± 17.5 years for female subjects. As advised by the general physician, all the subjects were suggested to take the *M. sylvestris* supplement daily. The measurements were carried out in three-time intervals (T0, T1, and T2), and included the bowel movements per week, VAS, and Bristol index. Table 4 shows the descriptive data (mean ± SD, minimum and maximum values) for male and female subjects at the three-time measurements.

#### 3.2.1. Primary and Secondary Objectives

**Figure 3:**
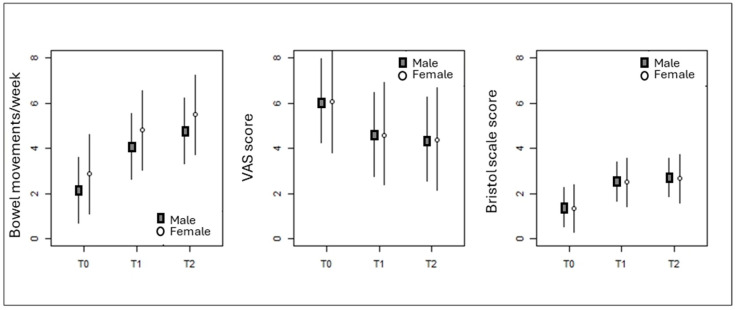
Trend of the three response variables in male and female subjects as a function of the three measurements as predicted (conditional effects) by the LMM models. The bars represent the 95% confidence intervals as predicted by a bootstrap (*n* = 10,000) on the variance–covariance matrix of the model coefficients for middle-aged individuals.

As reported in Table 5 and Figure 3, the LMM model for the number of bowel movements per week observed a significant effect for measurement (*p* < 0.001), but not by the age and sex of the subjects. In particular, the value of bowel movements per week increased significantly, both between T0 and T1 (β = 1.9 ± 0.2, t_110_ = 9.810, *p* < 0.001) and between T1 and T2 (β = 0.7 ± 0.2, t110 = 3.404, *p* < 0.001). Consequently, the difference between T0 and T2 is also statistically significant (β = 2.6 ± 0.2, t_110_ = 13.214, *p* < 0.001).

The LMM model for the VAS (Table 5 and Figure 3) identified significant effects for the measurement (*p* < 0.001) and for age (*p* = 0.04), but not for the sex of the subjects. The VAS score decreased significantly between T0 and T1 (β = 1.5 ± 0.2, t_110_ = 7.453, *p* < 0.001), but remained unchanged between T1 and T2 (β = 0.2 ± 0.2, t_110_ = 1.1167, *p* = 0.24). The difference between T0 and T2 remained significant (β = 1.7 ± 0.2, t_110_ = 8.621, *p* < 0.001). Regardless of the measurement, the VAS value decreased as the subject’s age increased (β = −0.035 ± 0.017, t_53_ = 2.098, *p* = 0.041).

The LMM model for the value of the Bristol scale (Table 5 and Figure 3) also identified a statistically significant effect for the measurement (*p* < 0.001), but not by the sex and age of the subjects. In particular, the value of the score increased significantly from T0 to T1 (β = 1.2 ± 0.1, t_110_ = 9.453, *p* < 0.001) and remained unchanged between T1 and T2 (β = 0.2 ± 0.1, t_110_ = 1.309, *p* = 0.19). Despite this, the difference between T0 and T2 was statistically significant (β = 1.3 ± 0.1, t_110_ = 10.761, *p* < 0.001).

#### 3.2.2. Tolerance and Satisfaction Assessment

To evaluate the tolerability of the *M. sylvestris* supplement, any adverse events were monitored throughout the period via spontaneous reporting by the participants to the general physician and pharmacist who performed the recruitment. During the 20-day period of taking the food supplement, no subject reported related adverse events, with the food supplement well-tolerated by the enrolled subjects.

## 4. Discussion

This study determined the effects of a food supplement based on a chemically characterized *M. sylvestris* extract for the management of FC by using self-assessment questionnaires on food supplement consumers, giving preliminary data regarding its efficacy and tolerability. The method used to perform this survey consisted of (i) a power analysis calculation, to determine the number of subjects involved in the survey; (ii) a definition of the inclusion and exclusion criteria; (iii) the use of validated questionnaires in the general population available in the Italian language to assess the frequency of bowel movements, stool consistency, and self-reported abdominal pain; and (iv) the use of inferential statistics to analyze the obtained results.

The results showed a significant improvement in bowel movements, with an average increase from 3.2 to 6.0 bowel movements per week in males and from 2.6 to 5.2 in females after using the supplement for 20 days. Stool consistency was improved as measured by the BSF scale, from hard and lumpy stools (type 1–2) to a ‘normal’ form (type 3–4). The VAS score indicated a marked reduction in the abdominal pain of participants. In addition, the supplement was well-tolerated and the participants’ satisfaction was good. As far as the chemical characterization of the main ingredient of the food supplement is concerned, *M. sylvestris* extract included both soluble and insoluble dietary fiber and other bioactive compounds from different chemical classes. In particular, there were many polyphenols, which, being poorly absorbed, remain for the most part in the gut where they become available for fermentation by the gut microbiota and are known to positively modify the gut microbiota to facilitate the maintenance of gut health, for example, favoring *Bifidobacterium* and *Lactobacillus* while inhibiting the growth of potentially pathogenic bacteria [28]. Moreover, the food supplement also contained sorbitol (10 g per daily dose—20 mL), present at a non-laxative effect dose, which is not digested or absorbed in the small intestine and holds water in its molecules, leading to increased water in the gut lumen, which could improve stool consistency. In addition, sorbitol is known to be fermented by the gut microbiota, increasing SCFA production [28].

Studies carried out over 50 years have revealed the presence of polysaccharides including mucilage in *M. sylvestris*, which possesses several biological properties and makes *M. sylvestris* extract a promising ingredient for food supplements and functional foods. The literature also indicates the presence of other phytochemicals in *M. sylvestris* extract such as amino acids (alanine, glycine, asparagine, glutamine, arginine, threonine, trigonelline, serine, and hydroxyproline), polyphenols (malvidin, malvin, delphidin, myricetin, apigenin, quercetin, kaempferol, genistein, phenolic acid derivatives, and coumarins), terpenoids (including carotenoids) [29,30]. The results of the current study confirmed the presence of polysaccharides in the *M. sylvestris* extract as indicated via the TDF content. Polysaccharides and mucilage are reported to increase the water content and weight of feces, enhance peristaltic movements, and speed up the gastric transit time. They may also restore hormones associated with the normal movements of the digestive tract [31,32]. Indirectly, mucilage possesses prebiotic actions while enhancing the growth of eubiotic bacteria strains and supporting overall health and wellbeing [33]. As revealed by the RP-UHPLC-MS analysis, *M. sylvestris* extract also contains phenolic acids, flavonoids, lipids, glycerides, amides, carotenoids, and chlorophyll derivatives (Table 1 and Table 2), showing the diversity of bioactive compounds in the extract, which can contribute synergistically to potential benefits for gastrointestinal health. Phenolic compounds may support gastrointestinal health and improve constipation by increasing intestinal transit and colon water content, as well as by modulating the gut microbiota [28,34,35,36]. Observational studies showed a direct link between dietary carotenoid intake and the risk of constipation, as an increased intake of dietary carotenoids resulted in a decreased risk of chronic constipation in American adults [37,38]. Data from the National Health and Nutrition Examination Survey (NHANES) demonstrated a negative correlation between increased dietary antioxidant intake and the prevalence of constipation [39]. In addition, an inverse relation between dietary lipids and total fats with constipation has been established by Rollet et al. [40]. Dietary lipids may regulate bowel movements via the increased colonic myoelectric and motor activity [41]. Flaxseed, a rich dietary source of polyunsaturated fatty acids (mainly α-linoleic acid), was observed to improve constipation symptom scores in constipated patients in a randomized clinical trial [42]. Another study demonstrated the effectiveness of the daily consumption of olive oil and flaxseed oil in the reduction of constipation symptom scores and the improvement of bowel movements and stool consistency [43]. However, it is worth mentioning that gastrointestinal problems including constipation are often associated with a high-fat diet in general [44], which suggests the laxative effects may not be attributed to all lipids but only some ‘healthy fats’.

The observed improvements in bowel movement frequency and stool consistency are consistent with earlier studies highlighting the role of mucilage and polyphenols in improving gastrointestinal transit. The ethnobotanical survey of *M. sylvestris* supports the effectiveness of its different components (leaves, flowers, aerial parts, roots, and whole plant) in managing abdominal pain and constipation, due to the presence of a high mucilage content [11]. Elsagh et al. [45] conducted a placebo-controlled trial to determine the efficacy of the aqueous extract obtained from *M. sylvestris* flowers on FC. Participants were randomized to receive either *M. sylvestris* flower extract (1 g/day) or a placebo for four weeks. The participants supplemented with *M. sylvestris* extract showed a significant increase in defecation frequency, improved stool consistency, and decreased overall constipation symptoms, suggesting *M. sylvestris* extract as a safe and effective preventive strategy in adults with FC. A study by Sleiman and Daher [12] demonstrated the anti-inflammatory and anti-ulcerogenic effects of *M. sylvestris* extract (500 mg/kg body weight) in a murine model by offering protection against gastric ulcers, which may support the improvement of abdominal pain with *M. sylvestris* supplementation as observed in our study. Esteves et al. [46] reported promising anti-nociceptive effects of *M. sylvestris* extract against acetic acid-induced abdominal constrictions and a capsaicin-induced pain model in mice. Another study demonstrated the protective effects of *M. sylvestris* extract and its polysaccharides against experimental ulcerative colitis in rats, by reducing the signs of inflammation [47].

This survey has its strengths, as the method used to perform this survey consisted of (i) a power analysis calculation, (ii) well-defined inclusion and exclusion criteria for the recruited population, and (iii) validated questionnaires and a statistical analysis for data interpretation. However, the results of this survey are considered preliminary as it is not a clinical trial, which would use a placebo control, blinding, and randomized approach with a relatively longer study duration. The lack of a placebo and the small sample size could limit the ability to definitively attribute the observed effects of the food supplement while the lack of blinding and a randomized approach could increase bias and influence the objectives as participants knew they were taking an active ingredient. No control over other dietary or lifestyle factors could also increase the potential confounders, that could impact constipation. Moreover, the self-reporting of objectives could increase the likelihood of inaccuracies and bias in subjective symptoms.

## 5. Conclusions

This survey-based study demonstrated that a *M. sylvestris*-based food supplement can improve intestinal function in individuals with FC by increasing bowel movements, improving stool consistency, and reducing abdominal pain. These findings support the potential of *M. sylvestris* supplementation as an effective alternative therapy for managing FC, particularly in individuals seeking non-pharmaceutical interventions.

The practical applications of these findings extend to clinical settings where nutritional advice could include *M. sylvestris*-based supplements as part of personalized dietary recommendations. By addressing FC symptoms and improving overall gut health, such supplements may serve as a complementary approach to traditional laxative therapies, especially for patients with mild to moderate FC. Future research should focus on validating these findings through randomized, double-blind, placebo-controlled trials with larger sample sizes and longer durations. These studies should also compare the efficacy of *M. sylvestris* supplements with conventional laxatives and explore their role in broader gastrointestinal health management. Additionally, further investigation into the bioactive compounds of *M. sylvestris* and their pharmacological effects could provide insights into its potential therapeutic applications beyond FC. By expanding on the clinical implications and exploring new therapeutic avenues, *M. sylvestris* could become a key component in the development of innovative and natural approaches to gastrointestinal health.

## Figures and Tables

**Table 1:** Identified compounds in *M. sylvestris* extract in negative ionization mode.

Peak	Rt	Compound	[M-H]^−^	MS/MS	Molecular Formula	Error (ppm)
1	0.78	Malic acid	133.05	115.0035; 71.0137	C_4_H_6_O_5_	4.3
2	4.35	Gallic acid	169.0142	125.0244; 79	C7H_6_O_5_	5.8
3	4.81	Vanillin (4-methoxybenzoic acid)	151.0401	136.0163; 107.0501	C_8_H_8_O_3_	6.06
4	5.33	Caffeic acid	179.0346	135.0449; 134.0375	C_9_H_8_O_4_	4.2
5	5.53	Rosmarinic acid	359.0772	135.0452	C_18_H_16_O_8_	0.67
6	6.2	Vicenin 2	593.1505	353.0660; 473.1084; 383.0766; 503.1191	C_27_H_30_O_15_	0.7
7	6.26	*p*-Coumaric acid	163.0401	119.0502; 91.0555	C_9_H_8_O_3_	0.8
8	6.36	Myricetin-3-hexoside-glucuronide	655.1152	479.0824; 316.0220	C_27_H_28_O_19_	−1.02
9	6.7	Quercitrin	447.0933	327.0507; 357.0609	C_21_H_20_O_11_	−0.67
10	7.07	Apigenin-4′-*O*-β-glucopyranoside	431.0984	269.0472; 311.0557; 341.0664	C_21_H_20_O_10_	−0.81
11	7.11	4-hydroxybenzoic acid	137.0242	93.0344	C_7_H_6_O_3_	2.3
12	7.53	Azelaic acid	187.0972	125.0970; 169.0869; 97.0657	C_9_H_16_O_4_	0.8
13	7.85	Gossypetin	317.0303	299.0194; 271.0243; 194.9932	C_15_H_10_O_8_	1.87
14	8.11	Myricetin-3-*O*-glucuronide	493.0616	317.0298	C_21_H_18_O_14_	0.74
15	8.17	Protocatechuic acid	153.0193	109.0294	C_7_H_6_O_4_	5.88
16	8.38	Quercetin-3-*O*-glucuronide	477.0675	301.035	C_21_H_18_O_13_	0.98
17	8.47	Rubone	373.1293	343.1179; 340.0942; 311.0916; 355.1180	C_20_H_22_O_7_	2.93
18	8.59	Kaempferol-3-O-glucuronide (orluteolin)	461.0725	285.0401	C_21_H_18_O_12_	2.09
19	8.65	Chysin	253.0502	143.1028; 119.0815	C_15_H_10_O_4_	1.69
20	8.65	Fisetin	285.0399	135.3614	C_15_H_10_O_6_	1.94
21	8.89	Quercetin	301.0354	178.9985; 151.0033	C_15_H_10_O_7_	2.31
22	9.19	Luteolin	285.0402	151	C_15_H_10_O_6_	2.26
23	9.57	Kaempferide-3-glucuronide	475.0898	299.0558; 284.0320	C_22_H_20_O_12_	5.77
24	9.59	Naringenin	270.95	151.0034; 119.0503; 107.0135	C_15_H_12_O_5_	2.66
25	9.73	Hesperetin	301.0718	151.0036; 178.9983	C_16_H_14_O_6_	2.81
26	9.99	Apigenin/Genistein	269.0455	149.0242; 117.0363	C_15_H_10_O_5_	2.66
27	9.99	9,12,13-trihydroxy-octadecadienoic acid	327.2172	211.1336; 229.1442; 171.1025	C_18_H_32_O_5_	1.91
28	10.07	Kaempferol	285.0401	257.0454; 217.2520; 151.0031	C_15_H_10_O_6_	2.58
29	10.28	Rhamnetin	315.051	164.9829; 300.0273	C_16_H_12_O_7_	2.41
30	10.52	9,12,13-trihydroxy-octadecenoic acid	329.233	229.1444; 211.1339; 171.1025	C_18_H_34_O_5_	2.19
31	10.94	1-*O*-palmitoylglyceride	329.2697	229.1443; 211.1337; 171.1020	C_19_H_38_O_4_	1.91
32	13.97	9-hydroxy-10(E),12(Z),15(Z)-octadecatrienoic acid	293.2118	275.2013; 183.1389; 171.1025	C_18_H_30_O_3_	0.98

**Table 2:** Identified compounds in *M. sylvestris* extract in positive ionization mode.

Peak	Rt	Compound	[M-H]^+^	MS/MS	Molecular Formula	Error (ppm)
1	6.2	Vicenin 2	595.1641	379.0807; 409.0908; 325.0702; 457.1110	C_27_H_30_O_15_	−0.57
2	6.53	*N*-(9-oxodecyl) acetamide	214.1797	214.1798; 161.1321; 95.0853; 137.1322	C_12_H_23_O_2_N	−1.47
3	6.83	Loliolide	197.1167	197.1169; 179.1063; 135.1165; 107.0853	C_11_H_16_O_3_	−0.34
4	8.35	Quercetin-3-*O*-β-glucuronide (Miquelianin)	479.0808	303.0493; 141.8239; 53.2160	C_21_H_18_O_13_	−0.73
5	8.53	Quercetin-3-*O*-β-glucuronide (Miquelianin)	479.0806	303.0493; 141.8239; 53.2160	C_21_H_18_O_13_	−0.73
6	8.6	Kaempferol-3-*O*-glucuronide (orluteolin)	463.0858	287.0544; 152.0563; 72.5275	C_21_H_18_O_12_	−0.8
7	10.89	Dehydrophytosphingosine	316.2839	60.0443; 280.2631; 316.2847	C_18_H_37_O_3_N	−0.68
8	11.04	Dehydrophytosphingosine	316.2838	60.0443; 280.2631; 316.2847	C_18_H_37_O_3_N	−0.81
9	11.46	Phytosphingosine	318.2994	60.0442; 282.2787; 318.2996	C_18_H_39_O_3_N	−0.5
10	13.96	Stearidonic acid	277.2155	135.1166; 121.1010; 277.2161	C_19_H_28_O_2_	−0.66
11	15.01	LPC (16:0)	496.3388	184.0730; 104.1068; 478.3288	C_24_H_50_O_7_NP	−2.79
12	15.6	1-*O*-linolenoyl glyceride	353.2677	335.2576; 317.2469; 289.2525	C_21_H_36_O_4_	0.35
13	17.94	Oleamide	282.2784	247.2416; 265.2520	C_18_H_35_NO	−0.73
14	18.83	Pheophorbide a	593.2738	533.2534	C_35_H_36_O_5_N_4_	−2.31
15	19.19	Pheophorbide a (isomer)	593.274	533.2535	C_35_H_36_O_5_N_4_	−2.31
16	20.85	Erucamide (13-cis-docosenamide)	338.3408	303.3041; 321.3145	C_22_H_43_ON	−2

**Table 3:** Demographic characteristics of the population involved in the survey.

Characteristics	Values
Gender	
Males	12
Females	44
Age	
Males	58.1 ± 18.3
Females	52.2 ± 17.5
Ethnicity	Caucasian

**Table 4:** Descriptive statistics (mean, standard deviation, minimum, and maximum) for the three variables of response calculated for men and women across the three measurements.

Variable	Gender	T0	T1	T2
Bowel movements per week	Male (*n* = 12)	3.2 ± 1.9 (1–4)	5.5 ± 2.6 (1–10)	6 ± 2.5 (1–10)
	Female (*n* = 44)	2.6 ± 0.8 (1–4)	4.5 ± 1.7 (1–7)	5.2 ± 2.1 (1–11)
VAS	Male (*n* = 12)	4.2 ± 2.6 (0–8)	2.3 ± 2.2 (0–8)	2.3 ± 2.2 (0–8)
	Female (*n* = 44)	4.2 ± 2.7 (0–8)	2.8 ± 2.1 (0–8)	2.5 ± 1.9 (0–8)
Bristol	Male (*n* = 12)	2.2 ± 0.6 (1–3)	3.2 ± 0.8 (2–4)	3.2 ± 0.8 (2–4)
	Female (*n* = 44)	2.0 ± 1.1 (1–3)	3.2 ± 1.2 (1–7)	3.4 ± 1.2 (1–7)

**Table 5:** Results of LMM models.

Model		Gdl		F	*p*
EV/WK	Measurement	2	110	94.149	<0.001
	Sex	1	53	1.873	0.18
	Age	1	53	0.459	0.50
VAS	Measurement	2	110	43.743	<0.001
	Sex	1	53	0.001	0.98
	Age	1	53	4.402	0.04
Bristol	Measurement	2	110	68.958	<0.001
	Sex	1	53	0.026	0.87
	Age	1	53	2.311	0.13

## Data Availability

The data will be made available upon request.
